# Validity and reliability of an adapted questionnaire measuring knowledge, awareness and practice regarding familial hypercholesterolaemia among primary care physicians in Malaysia

**DOI:** 10.1186/s12872-020-01845-y

**Published:** 2021-01-19

**Authors:** Ahmad Baihaqi Azraii, Anis Safura Ramli, Zaliha Ismail, Suraya Abdul-Razak, Siti Fatimah Badlishah-Sham, Noor Alicezah Mohd-Kasim, Norsiah Ali, Gerald F. Watts, Hapizah Nawawi

**Affiliations:** 1 Department of Primary Care Medicine, Faculty of Medicine, Universiti Teknologi MARA (UiTM), Selayang Campus, Jalan Prima Selayang 7, 68100 Batu Caves, Selangor Malaysia; 2 Institute of Pathology, Laboratory and Forensic Medicine (I-PPerForM), Universiti Teknologi MARA (UiTM), Sungai Buloh Campus, Jalan Hospital, 47000 Sungai Buloh, Selangor Malaysia; 3 Department of Public Health Medicine, Faculty of Medicine, Universiti Teknologi MARA (UiTM), Sungai Buloh Campus, Jalan Hospital, 47000 Sungai Buloh, Selangor Malaysia; 4 Department of Pathology, Faculty of Medicine, Universiti Teknologi MARA (UiTM), Sungai Buloh Campus, Jalan Hospital, 47000 Sungai Buloh, Selangor Malaysia; 5 Klinik Kesihatan Masjid Tanah, 78300 Masjid Tanah, Melaka Malaysia; 6 School of Medicine, Faculty of Health and Medical Sciences, University of Western Australia, GPO Box X2213, Perth, WA 6827 Australia; 7 Lipid Disorders Clinic, Department of Cardiology, Royal Perth Hospital, Perth, WA 6827 Australia

**Keywords:** Familial hypercholesterolaemia, Knowledge, awareness and practice, Questionnaire validation, Primary care, Malaysia

## Abstract

**Background:**

Primary care physicians (PCP) play an important role in detecting Familial Hypercholesterolaemia (FH) early. However, knowledge, awareness and practice (KAP) regarding FH among Malaysian PCP are not well established, and there was no validated tool to assess their FH KAP. Thus, the aim of this study was to adapt an FH KAP questionnaire and determine its validity and reliability among Malaysian PCP.

**Methods:**

This cross-sectional validation study involved Malaysian PCP with ≥ 1-year work experience in the primary care settings. In Phase 1, the original 19-item FH KAP questionnaire underwent content validation and adaptation by 7 experts. The questionnaire was then converted into an online survey instrument and was face validated by 10 PCP. In Phase 2, the adapted questionnaire was disseminated through e-mail to 1500 PCP. Data were collected on their KAP, demography, qualification and work experience. The construct validity was tested using known-groups validation method. The hypothesis was PCP holding postgraduate qualification (PCP-PG-Qual) would have better FH KAP compared with PCP without postgraduate qualification (PCP-noPG-Qual). Internal consistency reliability was calculated using Kuder Richardson formula-20 (KR-20) and test–retest reliability was tested on 26 PCP using kappa statistics.

**Results:**

During content validation and adaptation, 10 items remained unchanged, 8 items were modified, 1 item was moved to demography and 7 items were added. The adapted questionnaire consisted of 25 items (11 knowledge, 5 awareness and 9 practice items). A total of 130 out of 1500 PCP (response rate: 8.7%) completed the questionnaire. The mean percentage knowledge score was found to be significantly higher in PCP-PG-Qual compared with PCP-noPG-Qual (53.5, SD ± 13.9 vs. 35.9, SD ± 11.79), t(128) = 6.90, p < 0.001. The median percentage awareness score was found to be significantly higher in PCP-PG-Qual compared with PCP-noPG-Qual (15.4, IqR ± 23.08 vs. 7.7, IqR ± 15.38), p = 0.030. The mean percentage practice score was significantly higher in PCP-PG-Qual compared with PCP-noPG-Qual (69.2, SD ± 17.62 vs. 54.4, SD ± 19.28), t(128) = 3.79, p < 0.001. KR-20 value was 0.79 (moderate reliability) and average Kappa was 0.796 (substantial agreement).

**Conclusion:**

This study has proven that the 25-item adapted FH KAP questionnaire is valid and reliable. It can be used to measure and establish FH KAP among PCP in Malaysia.

## Background

Familial Hypercholesterolaemia (FH) is a genetic condition characterised by severely raised low density lipoprotein cholesterol (LDL-c) that leads to atherosclerosis, resulting in an increased risk for premature coronary artery disease (CAD) [1, 2]. It is one of the most common forms of inherited conditions with an autosomal mode of inheritance [2]. Mutations in several genes such as *LDLR*, *APOB* and *PCSK9* have been strongly linked to FH [2]. Clinically, this condition presents in the form of either heterozygous FH (HeFH) or homozygous FH (HoFH). HeFH is more common with an estimated 70–90% of FH cases resulting from heterozygous pathogenic variants [2, 3]. HeFH accounts for 2–3% of CAD in individuals below 60 years of age [1, 3]. In contrast, most individuals with HoFH experience severe CAD by their mid-20 s and the rate of either death or coronary bypass surgery by the teenage years is very high [1, 3]. Early detection and treatment of FH through cholesterol-lowering therapies can effectively prevent premature CAD [4].

Globally, HeFH prevalence is found to range from 1 in 200 to 1 in 500 in various populations [5–7]. In Malaysia, the prevalence of clinically diagnosed FH has been reported at 1 in 100, which is one of the highest in the world [8]. Malaysia is currently made up of a population at around 32 million, thus 320,000 individuals are estimated to have HeFH [8]. However, like in most countries, the majority of these cases are still undiagnosed, resulting in lost opportunities to prevent premature CAD [9, 10]. This has undoubtedly contributed to the high prevalence of premature CAD among Malaysians which accounted for 10–15% of acute coronary syndrome (ACS) [11]. A recent national report found that the mean age of individuals with ACS at admission in Malaysia was 58.6 years old, of which 23.8% were under the age of 50 years [12]. This is younger compared with our Asian counterparts in neighbouring countries [12, 13].

Improving identification of FH, particularly in primary care, enables early treatment of these individuals which is crucial to reduce their risk of premature CAD [14]. In Malaysia, primary care physicians (PCP) are the front liners of the primary care service be it in the public or private sectors [15]. They manage common cardiovascular risk factors such as diabetes, hypertension and hypercholesterolaemia [16]. However, only a handful of Malaysian PCP have formal postgraduate (PG) primary care qualification, while the majority do not [15, 16]. The situation in Malaysia is similar to many other developing countries where doctors without primary care qualification are permitted to practice as PCP [17]. This is in contrast to some developed countries such as Australia and the United Kingdom (UK) where having PG training and qualification in primary care is mandatory.

Numerous studies have shown that there were gaps in knowledge, awareness and practice (KAP) of FH among PCP in various parts of the world [18–25], especially in developing countries [25]. Pang et al*.* recently assessed FH KAP among PCP in several Asia–Pacific countries in the ‘Ten Countries Study’ [25]. Their KAP have been found to be suboptimal where less than 50% of the PCP knew of any available FH clinical guidelines; and their knowledge of genetic inheritability, prevalence, criteria for diagnosis and risk of CAD in FH were also found to be low [25]. Addressing these gaps is essential for effective implementation strategies to improve management of FH among PCP [26].

The 19-item FH KAP questionnaire designed by Bell et al*.* in 2014 [18] was utilized to assess the FH KP among PCP in the FH ‘Ten Countries Study’ [25]. This questionnaire was initially developed with the aim to evaluate the KAP with regards to FH among PCP in Western Australia [18]. Since then, this questionnaire has been validated and tested in several populations including the UK [21, 22], India [23] and Saudi Arabia [24]. Although the FH ‘Ten Countries Study’ already included 219 Malaysian PCP [25], little is known regarding the gaps in KAP between PCP holding PG qualification compared to those who do not hold any PG qualifications. To further extend this finding, there was a need to adapt and validate this questionnaire among the Malaysian PCP, comparing the two groups. Therefore, the objectives of this study were to adapt the 19-item FH KAP questionnaire into the local setting and to determine its validity and reliability among the Malaysian PCP with and without PG qualification. The ultimate aim was to utilize this tool to improve awareness and skills among PCP as part of a national implementation strategy for improved detection and care of FH within the society.

## Methods

This study was conducted under the auspices of the FH ‘Ten Countries Study’ which examined key components of FH care around the Asia–Pacific Region [25]. It was conducted in two parts. The first part involved adapting and validating the FH KAP questionnaire; and the second part was determination of the FH KAP among PCP in Malaysia using the questionnaire that has been adapted and validated. This paper presents the detailed methods and findings of the first part of the study. The second part of the study and its results was already published in 2018 [27]. The sampling for the first and second parts was mutually exclusive i.e. participants who were recruited in the first part of the study were excluded from the second part.

### Study design and participants

This cross-sectional questionnaire validation study was conducted in two phases. Phase 1 was the content validation, adaptation and face validation of the questionnaire. Phase 2 was the field testing and psychometric evaluation.

This study was conducted between January 2016 and January 2017 among PCP in Malaysia which consisted of Medical Officers (MO), General Practitioners (GP) and Family Medicine Specialists (FMS). Those who had full registration status with the Malaysian Medical Council and have ≥ 1 year of work experience in primary care were included. The exclusion criteria included those who worked on a locum basis in primary care or doctors from other medical specialties apart from primary care medicine.

PCP holding postgraduate qualification (PCP-PG-Qual) was defined as doctors who have completed primary care training and hold official PG qualifications i.e. Master of Family Medicine, Graduate Certificate or Diploma in Family Medicine (GCFM / DFM), Member of the Royal College of General Practitioners, United Kingdom (MRCGP, UK) or Fellow of the Royal Australian College of General Practitioners (FRACGP). PCP who do not hold any postgraduate qualification (PCP-noPG-Qual) was defined as doctors with basic medical degree such as Bachelor of Medicine, and Bachelor of Surgery (MBBS) and Doctor of Medicine (MD).

**Fig. 1:**
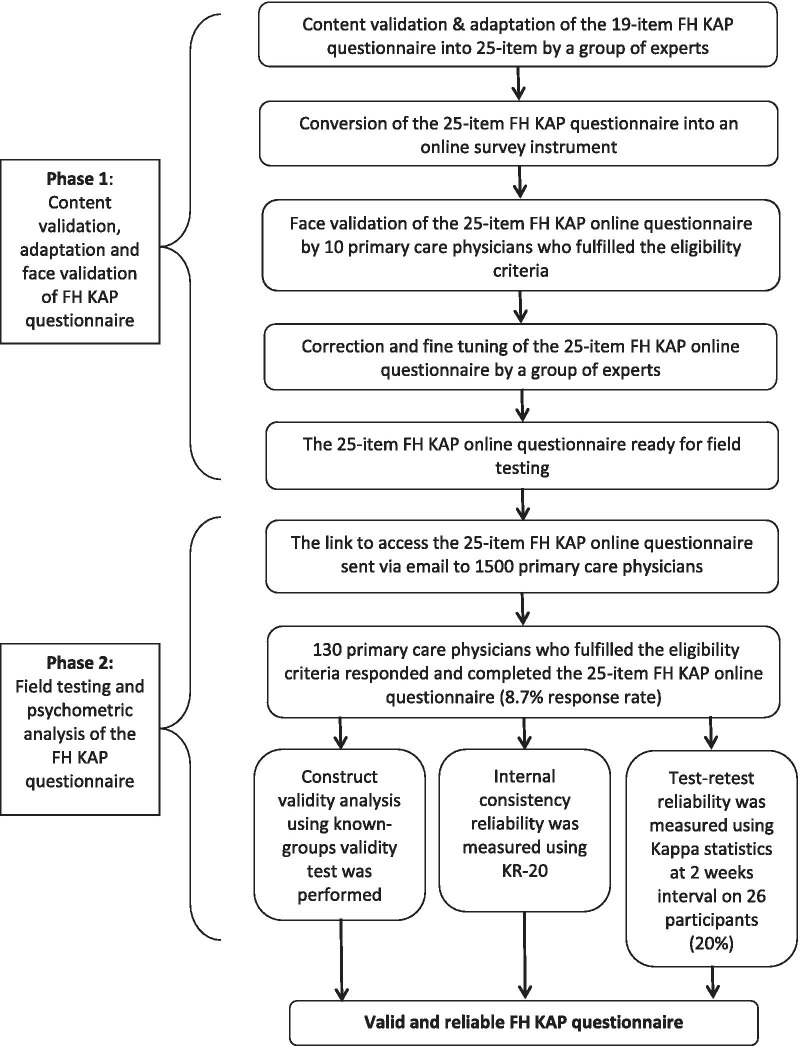
Flow chart of the conduct of the study

In Phase 2, the psychometric evaluation involved construct validity and reliability testing. Construct validity was conducted by testing differences between two groups with expected differences to establish known-groups validity [28]. The hypothesis was PCP-PG-Qual would have a better KAP regarding FH compared to PCP-noPG-Qual. Reliability testing involved internal consistency analysis using Kuder Richardson formula-20 (KR-20) reliability coefficient [29]; and test–retest reliability using Cohen’s kappa statistics to measure the stability of the responses to the questionnaire over time [30]. Figure 1 outlines the two phases of the adaptation and validation processes.

### Study instrument

Bell et al*.* developed the original FH KAP questionnaire used in this study [18]. Written permission to adapt and validate the questionnaire was obtained from the questionnaire developer via e-mail prior to the conduct of the study. The questionnaire is in the English language and consisted of 19 items divided into three domains i.e. knowledge, awareness and practice. The three domains in this questionnaire were derived from the Theory of Planned Behaviour (TPB) Model [31] and the Knowledge to Action (KTA) Framework [32]. The TPB model assumes that physician’s knowledge would affect physician’s awareness or attitude which would eventually affect physician’s behavior and practice. The KTA Framework incorporates the need to adapt the knowledge to fit with individual and the local context. Other pathways may be involved; however, these theoretical frameworks are thought to be the most appropriate to explain the domains in this questionnaire.

There were seven items in the knowledge domain covering the following areas: (i) description of FH; (ii) identification of lipid profile in FH; (iii) prevalence of FH in Australia; (iv) inheritance of FH in first-degree relatives; (v) CAD risk in untreated FH; (vi) age threshold for premature CAD; (vii) role of genetic testing in FH. The awareness domain consisted of three items covering the following areas; (i) familiarity with FH; (ii) Australian clinical guideline on FH; (iii) lipid specialist service. There were nine items in the practice domain covering the following areas; (i) assistance in FH detection; (ii) number of FH cases under care; (iii) screening of relatives in FH cases; (iv) family screening of FH among premature CAD; (v) preference on effective healthcare provider in FH detection; (vi) age for FH screening among young individuals in a family with premature CAD; (vii) referral of FH patients to lipid specialist; (viii) pharmacological agents used in hypercholesterolaemia; (ix) combination of pharmacological agents used in severe hypercholesterolaemia.

The questionnaire items and responses included 7-point Likert scale, single best answer, multiple answer, ‘Yes/ No/ Don’t know’ and free text answer. Most of the questions have predetermined correct answers.

### Phase 1: content validation, adaptation and face validation

#### Content validation

The content validation involved seven experts, consisting of three FMS who were accustomed to managing FH, three chemical pathologists with special interest in lipidology and one public health physician who was an expert in questionnaire validation. The panel of experts developed the conceptual framework for the FH KAP questionnaire based on the TPB model [31] and the KTA framework [32]. They then reviewed the original 19 items for conceptual and item equivalence. The panel of experts determined whether the contents of the questionnaire were relevant to the conceptual framework and the local context. They also reviewed the established clinical diagnostic criteria for FH i.e. the Simon Broome Register (SBR) Criteria [33], Dutch Lipid Clinic Network (DLCN) Criteria [34] and the United States (US) Make Early Diagnosis to Prevent Early Deaths (MED-PED) Criteria [35]. Clinical practice guidelines for management of FH were also reviewed by the panel of experts i.e. the UK National Institute for Health and Care Excellence (NICE) FH Guideline 2008 [36], International FH Foundation Guideline 2014 [37], European Atherosclerosis Society (EAS) FH Guideline 2014 [38], Japanese FH Guideline 2012 [39] and the American National Lipid Association (NLA) FH Guideline 2011 [40]. They reviewed the relevance of each questionnaire item against the current guideline recommendations including new treatment modalities. They also determined whether the number of items were sufficient to represent the questionnaire domains.

#### Adaptation of the questionnaire

**Fig. 2:**
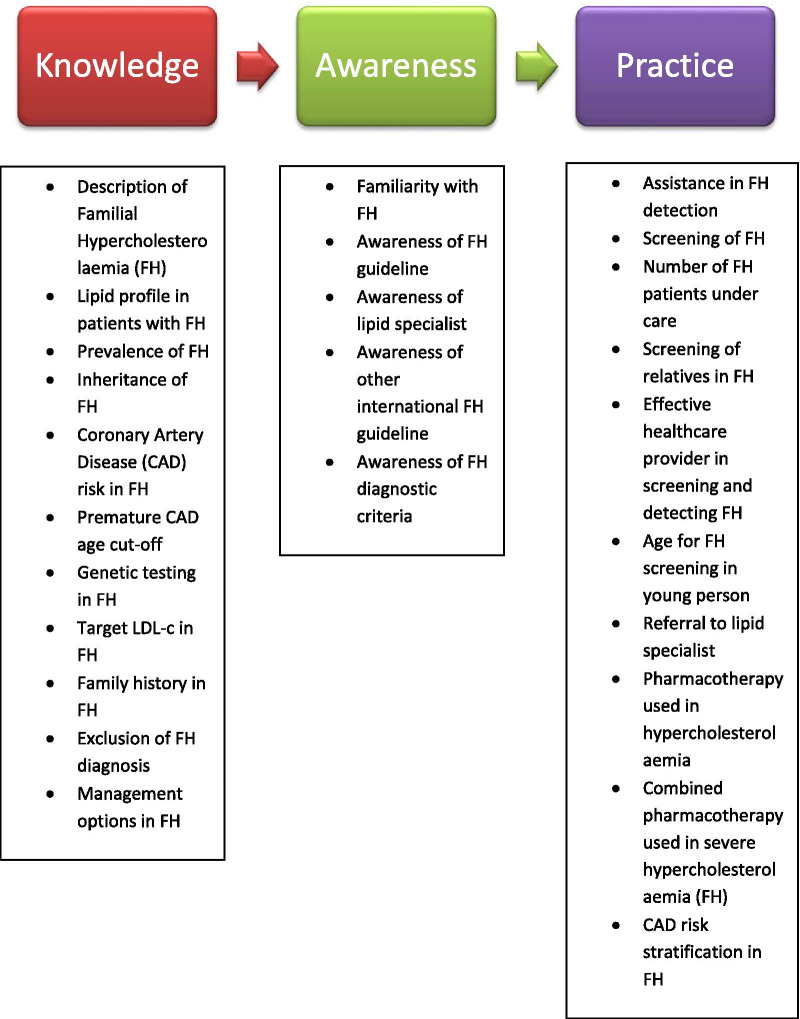
The conceptual framework of knowledge, awareness and practice regarding familial hypercholesterolaemia among primary care physicians in Malaysia

The FH KAP questionnaire was then adapted to include the three established clinical diagnostic criteria [33–35] and also recommendations by the current clinical practice guidelines [36–40] to ensure that it is up-to-date and usable. The questionnaire items were also adapted to suit the local context and items were added into the domains when the number was deemed insufficient to represent the domain. The questionnaire was not translated into the Malay language because most doctors in Malaysia including PCP are proficient in English. Figure 2 shows the conceptual framework of knowledge, awareness and practice of FH among primary care physicians in Malaysia and the items formation for each domain.

#### Conversion of the questionnaire into an online survey instrument

The adapted paper-based FH KAP questionnaire was then converted into an online survey questionnaire using the Google® Forms [41]. Relevant information about the study was given on the first page of the online survey and also in the email containing the link to the survey. The information given was the same as the paper-based information sheet, containing the identity of the researchers, contact details, the reason for conducting the survey and how the data would be used. Participants were informed that they have the right to pull out from the study at any time. The respondents were required to click the ‘Consent’ button as an indication that they have given an informed consent before the next page of the questionnaire could be accessed.

The questionnaire was divided into three sections. Section A contained questions on the inclusion and exclusion criteria. Participants could only proceed to Section B and C, if they fulfil the eligibility criteria. Section B contained questions covering the KAP domains. Section C included questions on demographic characteristic, qualification and work experience. Participants could omit to answer any of the items as none of them required an obligatory response (other than those relating to the consent) before they could proceed until the end of the questionnaire.

#### Face validation

Ten PCP who were naive to the study and fulfilled the inclusion and exclusion criteria were selected for face validation of the adapted FH KAP questionnaire. The 10 PCP were given both the paper-based and online versions of the questionnaire. The questionnaire was self-administered, and participants were requested to take note of the time taken to answer the questionnaire, clarity of the content, language and wording used and the general structure of the questionnaire. Their opinions on understanding the instructions and contents of the questionnaire were assessed and recorded. This included their understanding of the wording and general structure. Results were discussed among the panel of experts. Minor correction and fine tuning of the questionnaire were addressed according to their comments and suggestions.

### Phase 2: field testing and psychometric evaluation

The adapted FH KAP online questionnaire was field tested amongst PCP who fulfilled the same inclusion criteria as in Phase 1. However, PCP who participated in Phase 1 and 2 were mutually exclusive, as those who participated in Phase 1 were not recruited for Phase 2.

#### Sample size

Two sample sizes were calculated for this study, one for the known-groups validity and the other for the reliability testing. The known-groups validity compared mean or median percentage score of FH KAP between PCP-PG-Qual and PCP-noPG-Qual. Therefore, the sample size for each group was calculated using the OpenEpi software for comparison of two means formula [42]. As there was no previous study comparing KAP between PCP-PG-Qual and PCP-noPG-Qual related to FH, the sample size was therefore calculated based on the study by Mosli et al*.*, which compared KAP regarding colorectal cancer screening among two groups of PCP in Saudi Arabia [43]. Family Medicine trained physicians had higher mean knowledge score compared with physicians with MBBS only (4.93 ± 2.29 vs. 3.23 ± 1.88, P < 0.01) [43]. Based on the difference between the two means in this study, 95% Confidence Interval (2 sided), 80% power and a ratio of 1:1 between groups, the minimum sample size required for each group was 24 participants.

The sample size for the internal consistency reliability analysis using KR-20 was calculated based on the subject to item ratio for which a subject to item ratio of 5:1 was used [29]. Therefore, a minimum of 125 participants were needed (25 items × 5 = 125). Considering estimated response rate of 10–30% for an online survey [44], the questionnaire was planned to be distributed to at least 1500 participants. Regarding test–retest reliability, sample size for testing the Cohen’s kappa agreement was determined to be 26, which was 20% of the total number of participants [45].

#### Sampling method

A link to access the adapted FH KAP online questionnaire was sent via email to 1500 PCP in the e-mail lists of two major professional bodies for PCP in Malaysia. The email contained information on the study background, purpose and benefits, participation in the study, study procedure, confidentiality as well as informed consent. They were invited to open a link to the online questionnaire.

The questionnaire consisted of three sections as previously described. Those who consented to the study and fulfilled the study inclusion and exclusion criteria in Section A were able to proceed to Section B and C of the online form. Once the questionnaire was completed and submitted, no modification was allowed. To avoid repeated response from the same participant, the questionnaire contained an item asking whether they have answered the questionnaire previously. For test–retest reliability testing, those who have responded were contacted via e-mail to obtain their second response after two weeks of their first response.

#### Questionnaire interpretation, coding and scoring

For item no. 1 which assessed PCP’s familiarity with FH, scores of 5 to 7 were defined as ‘familiar’ and coded as ‘1′, while scores of 1 to 4 were defined as ‘unfamiliar’ and coded as ‘0′. The interpretation was done in accordance to the study by Rangarajan et al*.* [23] and Pang et al*.* [25]. For all of the items with predetermined correct answers, a correct response was coded as ‘1′, while an incorrect response was coded as ‘0′. The scoring of the questionnaire was determined based on the study by Batais et al*.* [24] by adding up the correct responses of the items in each domain. The minimum to maximum score for the knowledge domain ranged from 0 to 19, while the range for awareness domain was 0 to 13, and the range for practice domain was 0 to 9. Questions with multiple correct answers were scored accordingly for each correct response. Therefore, the maximum score for each domain was not the same as the number of items in that domain. The actual score for each domain was then transformed into a percentage score to make comparison between the domains easier to interpret. A score of ≥ 50% for each domain was considered acceptable [24, 25]. The scoring method of the adapted FH KAP questionnaire has already been provided in our previous publication [27].

#### Construct validation

The construct validity of the adapted FH KAP questionnaire was conducted using known-groups validation method because the questionnaire responses were dichotomous in nature rather than in numerical or continuous form [28]. The questionnaire would be considered valid if it is able to significantly discriminate across groups of subjects that have been predicted or ‘known’ to differ from each other [28]. In this study, the hypothesis made was PCP-PG-Qual would have a better KAP with regards to FH compared with PCP-noPG-Qual. Therefore, the FH KAP questionnaire would be considered valid if it could significantly discriminate the mean or median FH KAP scores between the two groups.

#### Reliability testing

Regarding the internal consistency reliability testing of the adapted FH KAP questionnaire, KR-20 reliability coefficient which is a special form of Cronbach’s alpha was carried out because the questionnaire’s responses were dichotomous in nature [29]. KR-20 reliability coefficient of < 0.50 was interpreted as low, 0.50—0.80 was considered moderate and > 0.80 was interpreted as high [29].

For the test–retest reliability, 26 participants (20%) were requested to answer the questionnaire again after two weeks interval. Cohen’s kappa statistics, which is a robust statistical method, was used for the reliability testing because of the dichotomous nature of the questionnaire responses [30]. The kappa result was interpreted as follows: values ≤ 0 indicated no agreement, 0.01—0.20 as none to slight, 0.21—0.40 as fair, 0.41—0.60 as moderate, 0.61—0.80 as substantial, and 0.81—1.00 as almost perfect agreement [30].

#### Statistical analysis

The SPSS software version 24.0 (SPSS Inc., Chicago, IL, USA) was used to analyse the data. Missing data were treated using a discrete value. Demographic background and practices information of the participants were analysed using descriptive statistics. Data were reported and presented as frequency and percentage. Before conducting the known-groups validity testing of FH KAP between PCP-PG-Qual and PCP-noPG-Qual, normality of data distribution was examined using visual inspection of the histogram frequency distribution and the Shapiro–Wilk Test. The Shapiro–Wilk Test was chosen as a supplementary test to the visual assessment of normality as this test is more appropriate for small sample size and is deemed to be the best choice for testing the normality of data [46]. A p-value of > 0.05 indicates that the data is normally distributed [46]. Levene's test for equality of variances was conducted to determine whether the variance in the two groups were homogeneous [47]. A p-value of > 0.05 for the F statistic indicates that the variances for the two groups are equal [47]. Independent *t*-test was used to compare the mean percentage score of FH KAP between the two groups for normally distributed data, whereas the Mann Whitney *u*-test was used for the non-normally distributed data. A p-value of < 0.05 was considered as statistically significant. For the internal consistency reliability testing, the KR-20 coefficient [48] for each KAP domain and the overall KR-20 coefficient were calculated. For the test–retest reliability testing using Cohen’s kappa statistics [30], the kappa value of each KAP item and the average kappa value were calculated.

## Ethical considerations

The ethical approval for this study was obtained from the Research Ethics Committee (REC), Universiti Teknologi MARA (600-IRMI (5/1/6). The designing of the online survey questionnaire using the Google® Forms complied with the British Psychological Society Ethics Guidelines for Internet-Mediated Research, 2013 [49]. The study information provided on the first page of the questionnaire emphasized that participants had the right to pull out from the study at any time. Informed consent was acquired via the online questionnaire when the respondent had to click the ‘Consent’ button before the next page of the questionnaire could be accessed. Participants could omit to answer any of the items as there was no item (other than those relating to the consent) required an obligatory response before they could proceed until the end of the questionnaire. The survey was made anonymous by switching the option to collect computer IP addresses to ‘No’. To ensure confidentiality, the password to the Google® Forms account was only known to the researcher and data were not stored within a shared account.

## Results

### Content validation, adaptation and face validation

During the content validation and adaptation process, a consensus decision was made by the panel of experts whereby 10 items remained unchanged, one item needed to be moved to the demography section, eight items were modified or rephrased to suit the local primary care settings and to ensure better understanding among Malaysian PCP; and seven new items were added. The additional items assessed wider areas of the FH KAP and increased the breadth of the questionnaire. The adapted and face validated FH KAP questionnaire consisted of 25 items and it remained in the English language. Table 1 summarizes the adaptation and modification made to the original 19-item FH KAP questionnaire. Further detail of the adaptation is provided in Additional file 1.

**Table 1:** Summary of adaptation of the FH KAP questionnaire

Item No	Original 19-Item FH KAP Questionnaire	Adapted 25-Item FH KAP Questionnaire
Knowledge domain		
3	Description of FH	Question rephrased
4	Lipid profile in FH	Answer rephrased
6	Prevalence of FH in Australia	Modified to prevalence of FH globally
7	Inheritance of FH	No change
8	CAD risk in FH	No change
9	Age threshold for premature CAD	Question rephrased
11	Genetic test in FH	No change
22		Added: Target LDL-c in FH
23		Added: Family history in FH
24		Added: Exclusion of FH diagnosis
25		Added: Management options in FH
Total items: 7		Total items: 11
Awareness domain		
1	Familiarity with FH	No change
2	Awareness of Australian FH guideline	Modified to awareness of NICE FH guideline
16	Awareness of lipid specialist service	No change except for question number
19		Added: Awareness of FH diagnostic criteria
20		Added: Awareness of other FH guidelines
Total items: 3		Total items: 5
Practice domain		
5	Assistance in FH detection	No change
10	Family screening of FH	Question rephrased
12	Number of FH patients under care	Moved to demography
13	Screening of relatives in FH	No change
14	Effective healthcare provider in FH	No change
15	Age for FH screening in young individuals	No change
17	Referral to lipid specialist	No change except for question number
18	Pharmacotherapy used in hypercholesterolaemia	Question rephrased
19	Combined pharmacotherapy used in severe hypercholesterolaemia	Question rephrased
21		Added: CAD risk stratification in FH
Total items: 9		Total items: 9

*FH*, familial hypercholesterolaemia; *CAD*, coronary artery disease; *LDL-c*, low density lipoprotein cholesterol; *NICE*, National Institute for Health and Care Excellence

Ten PCP face validated the paper-based and the online versions of questionnaire. They found that the questionnaire items were relevant in terms of content and were appropriately placed in the three domains. The language and wording used was clear and general structure and layout of both paper-based and the online versions were attractive. The time taken to answer the questionnaire was approximately 15 min. Minor correction and fine tuning of the questionnaire was done by the panel of experts, taking into consideration the comments and suggestions made by the PCP.

### Field testing and psychometric analysis

Out of 1500, a total of 130 (8.7%) PCP who fulfilled the eligibility criteria responded and completed the online questionnaire. The demographic and practice details of the participants are shown in Table 2. Out of 130 participants, PCP-PG-Qual consisted of 31 (23.8%) participants while PCP-noPG-Qual consisted of 99 (76.2%) participants. Both groups were homogeneous in terms of gender distribution, employer and practice locations. The main difference between the two groups was their medical qualifications. Out of 31 PCP-PG-Qual, 15 (48.4%) had DFM, 15 (48.4%) had Master of Family Medicine, and 1 (3.2%) had FRACGP certification. Majority (70.7%) of the PCP worked in urban areas.

**Table 2:** Demographic characteristics and practice details of the study participants

Characteristics of participants	PCP-PG-Qual n (%) 31 (23.8)	PCP-noPG-Qual n (%) 99 (76.2)	Total PCP n (%) n = 130 (100)	
*Gender*				
Male	7 (22.6)	30 (30.3)	37 (28.5)	
Female	24 (77.4)	69 (69.7)	93 (71.5)	
*Practice*				
Ministry of Health	15 (48.4)	56 (56.6)	71 (54.6)	
Ministry of Defence	0	2 (2)	2 (1.5)	
University	10 (32.3)	22 (22.2)	32 (24.6)	
Private services	6 (19.4)	19 (19.2)	25 (19.2)	
*Location*				
Rural	4 (12.9)	29 (29.3)	33 (25.4)	
Urban	27 (87.1)	70 (70.7)	97 (74.6)	
*Qualification*				
Basic medical degree (only)	0	99 (100)	99 (76.2)	
Diploma in Family Medicine	15 (48.4)	0	15 (11.5)	
Master of Family Medicine	15 (48.4)	0	15 (11.5)	
Fellow of Royal Australian College of GP	1 (3.2)	0	1 (0.8)	
Member of Royal College of GP (UK)	0	0	0	
*Service Duration*				
< 5 years	9 (29)	20 (20.2)	29 (22.3)	
5–10 years	14 (45.2)	53 (53.5)	67 (51.5)	
> 10 years	6 (19.4)	24 (24.2)	30 (23.1)	
No response	2 (6.5)	2 (2)	4 (3.1)	
*Patients Seen per Month*				
< 500 patients	22 (71)	41 (41.4)	63 (48.5)	
500–1000 patients	5 (16.1)	37 (37.4)	42 (32.3)	
> 1000 patients	2 (6.5)	9 (9.1)	11 (8.5)	
No response	2 (6.5)	12 (12.1)	14 (10.8)	
*FH Patients Under Care*				
No patient	15 (48.4)	65 (65.7)	80 (61.5)	
1–4 patients	11 (35.5)	13 (13.1)	24 (18.5)	
> 4 patients	2 (6.5)	5 (5.1)	7 (5.4)	
No response	3 (9.7)	16 (16.1)	19 (14.6)	

*n*, number; *PCP-PG-Qual*, primary care physicians with postgraduate qualification; *PCP-noPG-Qual*, primary care physicians without postgraduate qualification; *FH*, familial hypercholesterolaemia; *GP*, general practitioners; *UK*, United Kingdom

### Construct validity using known-groups validation

Independent *t*-test was used to compare the mean percentage knowledge and practice scores as the data were normally distributed. Mann Whitney *u*-test was used to compare the median percentage awareness scores as the data were not normally distributed.

Regarding knowledge, the mean percentage score was significantly higher in PCP-PG-Qual (53.5, SD ± 13.9) compared with PCP-noPG-Qual (35.9, SD ± 11.79), t(128) = 6.90, p < 0.001. The difference in the mean percentage knowledge score between the two groups was 17.54 (95% CI: 12.52, 22.57). Regarding practice, the mean percentage score was significantly higher in PCP-PG-Qual (69.2, SD ± 17.62) compared to PCP-noPG-Qual (54.4, SD ± 19.28), t(128) = 3.79, p < 0.001. The difference in the mean percentage practice score between the two groups was 14.74 (95% CI: 7.05, 22.44). The results are shown in Table 3.

**Table 3:** Known-groups validity by comparing mean percentage scores in knowledge and practice regarding FH between PCP-PG-Qual and PCP-noPG-Qual

KAP domain	Mean (± SD) percentage score of PCP-PG-Qual n = 31	Mean (± SD) percentage score of PCP-noPG-Qual n = 99	Mean difference in percentage score (95% CI)	t value^a^ (df)^b^	p-value^c^
Knowledge	53.5 (13.99)	35.9 (11.79)	17.54 (12.52, 22.57)	6.90 (128)	< 0.001
Practice	69.2 (17.62)	54.4 (19.28)	14.74 (7.05, 22.44)	3.79 (128)	< 0.001

*Abbreviations: KAP*, knowledge, awareness and practice; *SD*, standard deviation; *df,* degree of freedom; *CI*, confidence interval; *n*, number; *PCP-PG-Qual*, primary care physicians with postgraduate qualification; *PCP-noPG-Qual*, primary care physicians without postgraduate qualification;

^a^ Independent *t*-test was used to compare means as the data were normally distributed. The null hypothesis was rejected as the calculated *t* value > critical *t* value

^b^ df: degrees of freedom of the critical value of *t*

^c^ Significant *p* < 0.05

Regarding awareness, the median percentage score was significantly higher in PCP-PG-Qual (15.4, IqR ± 23.08) compared with PCP-noPG-Qual (7.7, IqR ± 15.38), p = 0.030. The difference in the median percentage awareness score between the two groups was 7.69. The results are shown in Table 4.

**Table 4:** Known-groups validity by comparing the median percentage score in awareness regarding FH between PCP-PG-Qual and PCP-noPG-Qual

KAP Domain	Median (± IqR) percentage score of PCP-PG-Qual n = 31	Median (± IqR) percentage score of PCP-noPG-Qual n = 99	Median difference in percentage score	z-value^a^	p-value^b^
Awareness	15.4 (23.08)	7.7 (15.38)	7.69	2.17	0.030

*KAP*, knowledge, awareness and practice; *IqR*, inter quartile range; *n*, number; *PCP-PG-Qual*, primary care physicians with postgraduate qualification; *PCP-noPG-Qual*, primary care physicians without postgraduate qualification;

^a^ Mann Whitney *u*-test was used to compare medians as the data was not normally distributed. The null hypothesis was rejected as the *z* value was > 1.96

^b^ Significant *p* < 0.05

### Internal consistency reliability using Kuder Richardson formula-20

The KR-20 internal consistency reliability coefficient of the FH KAP questionnaire was 0.53, 0.76 and 0.61 for knowledge, awareness and practice domains, respectively. The overall KR-20 coefficient for the FH KAP questionnaire was 0.79 which indicated moderate reliability [29]. The internal consistency reliability results of the FH KAP questionnaire are shown in Table 5.

**Table 5:** Internal consistency reliability of the FH KAP questionnaire

Item No	Area of KAP regarding FH	KR-20	Overall KR-20
*Knowledge domain*			0.79
3	Description of FH	0.53	
4	Identification of lipid profile in FH		
6	Prevalence of FH globally		
7	Transmission of FH to first-degree relative		
8	Rate of CAD risk in untreated FH		
9	Age threshold for premature CAD		
11	Genetic test for diagnosis of FH		
22	Target LDL-c in FH		
23	Important family history in FH		
24	Exclusion of the diagnosis of FH		
25	Management options in FH		
*Awareness domain*			
1	Familiarity with FH	0.76	
2	Awareness of NICE clinical guideline		
15	Awareness of lipid specialist		
19	Awareness of other FH clinical guidelines		
20	Awareness of FH diagnostic criteria		
*Practice domain*			
5	Assistance in detection of FH	0.61	
10	Screening for FH in premature CAD		
12	Family screening in FH patients		
13	Most effective healthcare provider to detect FH and screen first-degree relatives		
14	Age of FH screening among young person		
17	Pharmacotherapy used for hypercholesterolaemia		
18	Combined pharmacotherapy used for severe hypercholesterolaemia		
21	CAD risk stratification in FH		

*FH*, familial hypercholesterolaemia; *CAD*, coronary artery disease; *LDL-c*, low density lipoprotein cholesterol; *NICE*, National Institute for Health and Care Excellence;

### Test–retest reliability using Cohen’s kappa statistics

Out of 130 participants, 26 (20%) participants completed the questionnaire again at two weeks interval. The kappa values were 0.818, 0.810 and 0.760 for knowledge, awareness and practice domains, respectively. The average kappa value for the FH KAP questionnaire was 0.796, which indicated substantial agreement [30]. The test–retest reliability results of the FH KAP questionnaire are shown in Table 6.

**Table 6:** Test-restest reliability of the FH KAP questionnaire

No	KAP Domain	Item Kappa value	Domain Kappa value	Average Kappa value
*Knowledge domain*				0.796
3	Description of FH	0.879	0.818	
4	Identification of lipid profile in FH	0.865		
6	Prevalence of FH globally	0.759		
7	Transmission of FH to first-degree relative	0.811		
8	Rate of CAD risk in untreated FH	0.755		
9 (a)	Age for premature CAD (males)	0.920		
9 (b)	Age for premature CAD (females)	0.923		
11	Genetic test for diagnosis of FH	0.650		
22	Target LDL-c in FH	0.854		
23	Important family history in FH	0.695		
24	Exclusion of the diagnosis of FH	0.869		
25	Management options in FH	0.835		
*Awareness domain*				
1	Familiarity with FH	0.819	0.810	
2	Awareness of NICE clinical guideline	0.769		
15	Awareness of lipid specialist	0.920		
19	Awareness of other FH clinical guidelines	0.648		
20	Awareness of FH diagnostic criteria	0.893		
*Practice domain*				
5	Assistance in detection of FH	0.877	0.760	
10	Screening for FH in premature CAD	0.641		
12	Family screening in FH patients	0.507		
13	Most effective provider in FH detection	0.622		
14	Age of FH screening among young person	0.685		
16	Referral to lipid specialist	1.000		
17	Pharmacotherapy used for hypercholesterolaemia	0.943		
18	Combined pharmacotherapy used for severe hypercholesterolaemia	0.692		
21	CAD risk stratification in FH	0.872		

*FH*, familial hypercholesterolaemia; *CAD*, coronary artery disease; *LDL-c*, low density lipoprotein cholesterol; *NICE*, National Institute for Health and Care Excellence;

This study has produced a valid and reliable 25-item FH KAP questionnaire, and the final version was provided in our previous publication [27].

## Discussion

To the best of our knowledge, this is the first study in Malaysia which has adapted a questionnaire to assess KAP among PCP to suit the local primary care setting. Malaysian PCP have great potential to play a key role in the early detection of FH and its management in the community. However, they would require adequate knowledge and awareness regarding FH in order to improve their practice [26]. Therefore, the novel adaptation and validation of the FH KAP questionnaire in this study was crucial in order to produce a valid and reliable tool to evaluate KAP with regards to FH among PCP in Malaysia.

The adapted and validated FH KAP questionnaire consisted of 11 knowledge items, 5 awareness items and 9 practice items, giving a total of 25 items. In comparison to the study by Batais et al., the same questionnaire was adapted among their population into 23 items which encompassed 9 knowledge items, 5 awareness items and 9 practice items [24].

Overall, 10 items remained unchanged, 1 item was moved to demography, 8 items were modified or rephrased and 7 new items were added during the content validation and adaptation process. With regards to the knowledge domain, 3 items remained unchanged, 1 item was modified, 3 were rephrased for clarity and 4 items were added, giving a total of 11 items. The item assessing knowledge on prevalence of FH in Australia was found to be irrelevant to our PCP population and was therefore modified to global prevalence of FH. The prevalence of FH in the Malaysian population was not known at the time of adaptation of this questionnaire in 2016, and therefore the panel felt that it was appropriate to assess the PCP knowledge on the global prevalence. The literature estimating the prevalence of clinically diagnosed FH at 1 in 100 was only published in 2018 [8]. Similar to the study in Saudi Arabia by Batais et al*.*, the same item was changed to the global FH prevalence as the FH prevalence in their population was also unknown [24]. The four additional items included knowledge on target LDL-c level in FH, knowledge on the importance of family history in FH, exclusion of FH diagnosis as well as options in the FH management. Items number 23 and 24 were added to test knowledge regarding the need to look for tendon xanthomata and arcus cornealis in patients with suspected FH and their relatives. These clinical signs are highly suggestive of FH as recommended by established FH diagnostic criteria i.e. SBR Criteria [33] and DLCN Criteria [34]. They were added to ensure broader coverage of FH knowledge assessment among Malaysian PCP.

Concerning the awareness domain, 2 items remained unchanged, 1 item was modified and 2 items were added, giving a total of 5 items. The item assessing awareness of the Australian FH guideline was modified to awareness of the UK NICE FH Guideline 2008 [36]. The experts perceived that the awareness of the NICE FH guideline was appropriate as Malaysian PCP were thought to be more familiar with the UK-based guideline in the absence of a local Malaysian guideline on FH. An item assessing the awareness of PCP of several other international FH guidelines was also added i.e. International FH Foundation Guideline 2014 [37], EAS FH Guideline 2014 [38], Japanese FH Guideline 2012 [39] and the American NLA FH Guideline 2011 [40]. The addition was considered important as it would broaden the scope of assessment of PCP’s awareness on other international FH guidelines apart from the NICE guideline. Another item to assess PCP’s awareness of FH diagnostic criteria i.e. SBR Criteria [33], DLCN Criteria [34] and the US MED-PED Criteria [35] was also added. This was crucial as these criteria would be useful in detection and diagnosis of FH, and would be relevant to our PCP. The SBR Criteria was identified to perform better in detecting FH cases among Malaysian population compared to DLCN and US MED-PED Criteria as evidenced by a recent local study by Abdul-Razak et al*.* [50].

Regarding the practice domain, 5 items remained unchanged, 1 was moved to demography, 3 items were rephrased for clarity and 1 item was added, giving a total of 9 items. The item assessing the number of FH patients under care was moved to demography, as it was perceived to fit better in the demographic section. An item assessing practice in CAD risk stratification among FH patients was added. This is crucial as FH is known to carry a high mortality risk from CAD thus it is pivotal to assess whether PCP would still risk stratify patients with FH. Risk stratification in patients with FH is not recommended as they should be classified as high risk irrespective of other risk factors [51].

With regards to construct validity, known-groups validation was conducted due to the dichotomous nature of the responses in the FH KAP questionnaire [28]. Exploratory factor analysis (EFA) which is commonly used for numerical or continuous responses items is not recommended to assess construct validity of a questionnaire which is dichotomously scored [28]. In this study, PCP-PG-Qual had significantly higher mean percentage scores for knowledge and practice compared with PCP-noPG-Qual. Similar trend was also found with regards to the median percentage score for awareness. This is in keeping with the hypothesis of this study where PCP-PG-Qual was expected to perform better in all KAP domains. This finding indicated that the adapted 25-item FH KAP questionnaire was a valid tool to be used for determination of FH KAP among PCP in Malaysia. Furthermore, the second part of our study which had used the 25-item adapted and validated questionnaire to determine the FH KAP among Malaysian PCP showed considerable gaps between the two groups, where PCP-PG-Qual were found to have better knowledge, awareness and practice than PCP-noPG-Qual [27]. PCP-PG-Qual more often have patients with FH under their care. Therefore, the increased knowledge, awareness and practice could be due to these PCP already treating FH patients.

Regarding internal consistency, the KR-20 reliability coefficient which is an alternative to Cronbach’s alpha was carried out in this study because of the dichotomous nature of the questionnaire responses [29]. The KR-20 internal consistency coefficients for all the three domains showed moderate reliability (knowledge: 0.53, awareness: 0.76 and practice: 0.61). The overall KR-20 for the FH KAP questionnaire also showed moderate reliability at 0.79 [29]. Direct comparison with other studies assessing the FH KAP questionnaire could not be made as none of the other studies used KR-20 internal consistency reliability. Batais et al*.* reported Cronbach’s alpha value of > 7.0 instead of KR-20 in their study [24]. However, the use of this particular method was not justified by the authors [24].

Regarding test–retest reliability analysis, Cohen’s kappa coefficient was appropriately used instead of intra-class correlation coefficient (ICC) because of the dichotomous nature of the questionnaire responses [30]. Cohen’s kappa statistic is a form of reliability coefficient which is used to determine the degree of agreement between two different evaluations in questionnaires with dichotomous variables [30]. In this study, the kappa values were almost perfect for the knowledge (0.818) and awareness (0.810) domains while the practice domain showed substantial agreement (0.760). The average kappa coefficient of the FH KAP questionnaire was 0.796, indicating substantial agreement [30]. Therefore, this questionnaire was considered reliable and stable over time to be used among Malaysian PCP. In comparison to the study by Batais et al., they reported an average kappa of 0.85 [24], which was comparable to our study.

## Strengths and limitations

The strength of this study included the robust adaptation and validation methods used to determine the validity and reliability of this questionnaire among the Malaysian PCP. The 25-item questionnaire is also more comprehensive than the original 19-item questionnaire as it covers a wider scope of FH care. These include areas such as awareness of various FH diagnostic criteria and practice on CAD risk stratification for patients with FH.

There were several inevitable limitations in this study which include the low response rate, which is expected for an online questionnaire disseminated through emails [44]. The low response rate may lead to nonresponse bias. However, despite the low response rate, this study was able to achieve adequate sample size for psychometric evaluation to ensure validity and reliability of the questionnaire. Another limitation was the possibility of response bias in this study. PCP who answered the questionnaire may be those who have more awareness and knowledge of dyslipidaemia. The use of online questionnaire was also vulnerable to response bias in which the PCP could get the information easily from the internet. We also did not perform item-level content validity index [I-CVI] in this study, which would have provided objective measurements of content validity for this questionnaire [52]. However, the panel of experts reviewed the relevance of each item in the questionnaire and determined whether the contents were fit for the conceptual framework, questionnaire domains, current clinical practice guidelines and the local context. This intuitive exercise by panel of experts is also an acceptable method of content validation to ensure that the items of an instrument are relevant and sufficiently represent the content domains [53]. The scale-level content validity index [S-CVI] was also not performed as most of the items in this questionnaire have dichotomous responses with predetermined correct answers, rather than Likert scale. EFA was also not performed to assess the construct validity of FH KAP as most of the items were dichotomously scored [28]. Confirmatory factor analysis [CFA] was not performed in this study due to the need for a larger sample size and the limited time frame given to complete the study. Measuring the criterion validity i.e. comparing the score of a questionnaire with another questionnaire [53] is also beyond the scope of this study.

### Implications for future research and clinical practice

This study has produced a valid and reliable paper-based as well as online version of the questionnaire which can be used to evaluate the KAP with regards to FH among PCP in Malaysia. However, it is recommended that researchers should at least perform the KR-20 internal consistency analysis to ensure that the questionnaire is reliable in their respective populations. I-CVI should also be performed to provide objective measurements of the content validity for this questionnaire. Future research may also include CFA to assess the psychometric performance of the FH KAP questionnaire and criterion validation to compare its scoring with another FH KAP questionnaire. To ensure representative and unbiased sampling, future research should be conducted using a multi-stage random sampling. Future revision to the questionnaire may include testing knowledge and understanding of the PCP on the clinical diagnostic criteria of FH and how the co-presence of metabolic and secondary causes of dyslipidaemia may affect the diagnosis and management. It is indeed important for PCP to differentiate between FH and other metabolic comorbidities causing dyslipidaemia such as diabetes. A qualitative study to explore facilitators and barriers with regards to FH care in the Malaysian primary care settings should also be considered. Further research should also evaluate the resource implication to improve FH care in the Malaysian society including the pathways of referral for genetic testing. All of these research evidence are pertinent to support development of a national FH clinical practice guideline and FH training module for the Malaysian PCP.

## Conclusion

This study has produced a valid and reliable 25-item questionnaire which can be used to measure FH KAP among PCP in Malaysia. This would allow us to identify gaps in KAP regarding FH among those with and without PG qualifications in primary care. Subsequently, national FH clinical practice guideline and training module to address these gaps among Malaysian PCP should be developed. These strategies are vital if we were to improve our clinical management of FH in primary care, which in turn would enhance opportunities for premature CAD prevention.

## Supplementary information

**Additional file 1.** Adaptation of the FH KAP Questionnaire.

## Data Availability

Data are kept at the Institute of Pathology, Laboratory and Forensic Medicine (IPPerForM), Universiti Teknologi MARA (UiTM), Sungai Buloh Campus, Jalan Hospital, 47,000 Sungai Buloh, Selangor, Malaysia. Data will be shared upon request and is subjected to the data protection regulations.

## Abbreviations

FH: Familial hypercholesterolaemia
LDL-c: Low density lipoprotein cholesterol
CAD: Coronary artery disease
HeFH: Heterozygous FH
HoFH: Homozygous FH
ACS: Acute coronary syndrome
PCP: Primary care physicians
PG: Postgraduate
UK: United Kingdom
KAP: Knowledge, awareness and practice
MO: Medical officers
GP: General practitioners
FMS: Family medicine specialists
PCP-PG-Qual: PCP with postgraduate qualification
DFM: Diploma in Family Medicine
FRACGP: Fellow of the Royal Australian College of General Practitioners
MRCGP, UK: Membership of the Royal College of General Practitioners, UK
PCP-noPG-Qual: PCP without postgraduate qualification
MBBS: Bachelor of Medicine, Bachelor of Surgery
MD: Doctor of Medicine
KR-20: Kuder Richardson formula-20
REC: Research Ethics Committee
SD: Standard deviation
df: Degree of freedom
CI: Confidence interval
IqR: Inter quartile range
NICE: National Institute for Health and Care Excellence
EAS: European Atherosclerosis Society
NLA: National Lipid Association
SBR: Simon Broome Register
DLCN: Dutch Lipid Clinic Network
US: United States
MED-PED: Make Early Diagnosis to Prevent Early Deaths
I-CVI: Item-level content validity index
S-CVI: Scale-level content validity index
EFA: Exploratory Factor Analysis
CFA: Confirmatory Factor Analysis
