# Supplementary material for: Validity and reliability of an adapted questionnaire measuring knowledge, awareness and practice regarding familial hypercholesterolaemia among primary care physicians in Malaysia
Source: BMC Cardiovasc Disord. 2021 Jan 19;21:39. doi: 10.1186/s12872-020-01845-y (PMC7814747; doi:10.1186/s12872-020-01845-y)
Supplement: Supplementary file 1 — Additional file 1. Adaptation of the FH KAP Questionnaire. [file 12872_2020_1845_MOESM1_ESM.pdf]

Supplementary File 1: Adaptation of the FH KAP Questionnaire

Adaptation of the FH KAP Questionnaire for Knowledge Domain

Item No.	Area	Original Question and Answer	Modification/ Addition
3	Description of FH	Question:<br><br>Which one description below best describes FH?<br><br>Answer:<br><br>- The presence of family members with diagnosed high cholesterol<br>- A genetic disorder that is characterized by very high cholesterol and a family history of premature heart disease<br>- The presence of multiple lipid abnormalities that may be genetic in nature<br>- An ultra-rare, potentially fatal condition caused by cholesterol levels that can be up to six times the normal level<br>- Don`t know<br>- Others (please specify)	Question: Rephrased<br><br>Which one of the following descriptions best describes FH?<br><br>Answer: No change
4	Lipid profile of FH	Question:<br><br>Which one of the following lipid profiles is most consistent with the diagnosis of FH? (Reference intervals: Total cholesterol < 5.5mmol/L; Triglyceride < 1.7mmol/L; HDL-cholesterol >1.0mmol/L; LDL-cholesterol < 3.5mmol/L)	Question: No change

		Answer:<br><br>- Total cholesterol 6.0mmol/L; Triglyceride 3.4mmol/L; HDL-cholesterol 0.8mmol/L; LDL-cholesterol 3.8mmol/L<br>- Total cholesterol 6.3mmol/L; Triglyceride 12.2mmol/L; HDL-cholesterol 1.0mmol/L; LDL- -mmol/L<br>- Total cholesterol 8.0mmol/L; Triglyceride 1.1mmol/L; HDL-cholesterol 1.0mmol/L; LDL-cholesterol 6.5mmol/L<br>- Total cholesterol 5.4mmol/L; Triglyceride 1.3mmol/L; HDL-cholesterol 1.7mmol/L; LDL-cholesterol 3.1mmol/L<br>- Total cholesterol 7.1mmol/L; Triglyceride 1.0mmol/L; HDL-cholesterol 3.5mmol/L; LDL-cholesterol 3.2mmol/L	Answer: Rephrased<br><br>- Total cholesterol 6.3mmol/L; Triglyceride 12.2mmol/L; HDL-cholesterol 1.0mmol/L; LDL-cholesterol not reported
6	Prevalence of FH	Question:<br><br>What is the prevalence of FH in Australia?<br><br>Answer:<br><br>- 1 in 100 persons<br>- 1 in 500 persons<br>- 1 in 1000 persons<br>- 1 in 2000 persons<br>- 1 in 5000 persons<br>- Don`t know	Question: Modified<br><br>What is the prevalence of FH globally?<br><br>Answer: No change

7	Inheritance of FH	Question:<br><br>What is the likelihood that first-degree relatives (i.e. parents, siblings and children) of someone who has FH will also have the condition themselves?<br><br>Answer:<br><br>- 0%<br>- 25%<br>- 50%<br>- 75%<br>- 100%<br>- Don`t know	Question: No change<br><br>Answer: No change
8	CAD risk in FH	Question<br><br>How much greater is the risk of premature coronary artery disease (CAD) in untreated FH patients compared to the general population?<br><br>Answer:<br><br>- 2 times greater<br>- 5 times greater<br>- 10 times greater<br>- 20 times greater<br>- 50 times greater<br>- Don`t know	Question: No change<br><br>Answer: No change
9	Premature CAD age	Question:<br><br>When you are assessing a patient's family history, at what age for males and females do you consider CAD to be 'premature'? Leave blank if you wish to answer 'Don`t know'	Question: Rephrased<br><br>When you are assessing a patient's family history, at what cut-off age for males and females do you consider CAD to be 'premature'? Leave blank if you wish to answer 'Don`t know'

11	Genetic test in FH	Question:<br><br>Is the following statement true or false? An accurate diagnosis of FH can only be made via genetic test.<br><br>Answer:<br><br>- True<br>- False<br>- Don`t know	Question: No change<br><br>Answer: No change
22	Target LDL-c in FH	*Nil	New Question:<br><br>What is the target LDL-c following maximum tolerated dose of high intensity statin for patients diagnosed with FH?<br><br>Answer:<br><br>- < 1.8 mmol/L<br>- < 2.6 mmol/L<br>- < 3.4 mmol/L<br>- > 50% reduction from baseline LDL-c concentration<br>- > 30% reduction from baseline LDL-c concentration<br>- Don`t know
23	Family history in FH	*Nil	New Question:<br><br>Which of the following options are important to obtain from family history in patients with FH? (Please select all that apply)<br><br>Answer:<br><br>- Consanguinity<br>- Family history of premature CAD (age of

			onset)<br>- Family history of hypercholesterolaemia (TC and/ or LDL-c)<br>- Family history of tendon xanthomata<br>- Family history of unexplained childhood death<br>- Three-generation pedigree chart<br>- None of the above<br>- Don`t know
24	Exclusion of FH diagnosis	*Nil	New Question:<br><br>Which of the following options would exclude the diagnosis of FH? (Please select all that apply)<br><br>Answer:<br><br>- Normal LDL-c concentration before puberty<br>- Absence of clinical signs (e.g. tendon xanthomata)<br>- Absence of mutation on genetic testing<br>- Absence of ultrasound finding of tendon xanthomata<br>- None of the above<br>- Don`t know
25	Management options in FH	*Nil	New Question:<br><br>Which of the following are the management options for FH patients? (Please select all that apply)<br><br>Answer:<br><br>- Lifestyle modification is a substitute for lipid lowering medication<br>- Ezetimibe co-administered with statin therapy

	is recommended as an option for adult heterozygous FH<br>- Lipid lowering drug therapy is considered by the age of 10 years<br>- Lipid modifying therapy should be continued during pregnancy<br>- Progress of cascade screening in FH patients should be recorded<br>- None of the above<br>- Don`t know
**Total items: 7**	**Total items: 11**

Adaptation of the FH KAP Questionnaire for Awareness Domain

Item No.	Area	Original Question and Answer	Modification/ Addition
1	Familiarity with FH	Question:<br><br>On a scale of 1 to 7; how familiar are you with FH? (1-Never heard about it to 7-very familiar)<br><br>Answer:<br><br>7-point Likert scale (score of 5,6,7 is considered familiar)	Question: No change<br><br>Answer: No change
2	Awareness of FH guideline	Question:<br><br>Are you aware of the National Heart Foundation/ Cardiac Society of Australian and New Zealand guidelines on the detection and management of Familial Hypercholesterolaemia?<br><br>Answer:<br><br>- Yes<br>- No	Question: Modified<br><br>Are you aware of the National Institute for Health and Care Excellence (NICE) on Identification and Management of Familial Hypercholesterolaemia (2008)?<br><br>Answer: No change
16	Awareness of lipid specialist	Question:<br><br>Are you aware of any specialist-clinical services for lipid disorders to whom you can refer patients?<br><br>Answer:<br><br>- Yes<br>- No (Go to question 18)	Question: No change<br><br>Answer: No change except for question number<br><br>- Yes<br>- No (Go to question 17)

			*This item was re-numbered to 15
19	Awareness of other international FH guideline	*Nil	New Question:<br><br>Which of the following FH clinical guidelines that you are aware of? (Please select all that apply)<br><br>Answer:<br><br>- Integrated Guidance on the Care of Familial Hypercholesterolaemia by the International FH Foundation (2014)<br>- Homozygous Familial Hypercholesterolaemia: New Insight and Guidance for Clinicians to Improve Detection and Clinical Management. A Position Paper from the Consensus Panel on Familial Hypercholesterolaemia of the European Atherosclerosis Society (EAS) (2014)<br>- Guidelines for the Management of Familial Hypercholesterolemia (Harada-Shiba, 2012)<br>- Clinical Guidance by the National Lipid Association (NLA) Expert Panel on Familial Hypercholesterolaemia (2011)<br>- None<br>- Others (please specify)
20	Awareness of FH diagnostic criteria	*Nil	New Question:<br><br>Which of the following FH diagnostic criteria that you are aware of? (Please select all that apply)<br><br>Answer:<br><br>- Dutch Lipid Clinic Network Criteria<br>- Simon Broome Register Criteria<br>- US MED-PED Criteria

	- Japanese Criteria<br>- None<br>- Others (please specify)
**Total items: 3**	**Total items: 5**

Adaptation of the FH KAP Questionnaire for Practice Domain

Item No.	Area	Original Question and Answer	Modification/Addition
5	Assistance in FH detection	Question:<br><br>Which one of the following options could usefully assist you in detection of FH in your practice?<br><br>Answer:<br><br>- Laboratory report on a lipid profile alerting possible FH<br>- Alert by the clinical software system in your practice<br>- Direct telephone call from the laboratory<br>- All of the above<br>- None of the above<br>- Don`t know<br>- Others (please specify)	Question: No change<br><br>Answer: No change
10	Family screening of FH	Question:<br><br>In patients with documented premature CAD, which of the following do you routinely carry out? (Please tick all that apply)<br><br>Answer:<br><br>- Look for arcus cornealis<br>- Look for tendon xanthomata<br>- Take a detailed family history of CAD<br>- Screen close relatives for hypercholesterolaemia<br>- All of the above	Question: Rephrased<br><br>In patients with documented premature CAD, which of the following options do you routinely carry out? (Please tick all that apply)<br><br>Answer: No change

		- None of the above	
12	Number of FH patients under care	Question:<br><br>How many patients currently under your care, if any, have been formally diagnosed with FH?<br><br>*Fill in answer	Moved to demography
13	Screening of relatives in FH	Question:<br><br>If you have patients with FH under your care, do you routinely screen close relatives for this condition with a lipid profile?<br><br>Answer:<br><br>- Yes, patient's children only<br>- Yes, patient's children and other close relatives<br>- No<br>- Not applicable	Question: No change<br><br>Answer: No change<br><br>*This item was re-numbered to 12
14	Effective healthcare provider in FH	Question:<br><br>In your view, which healthcare providers would be most effective at early detection of FH and screening first-degree relatives? (Please tick up to two options)<br><br>Answer:<br><br>- Lipid specialists<br>- General practitioners<br>- Cardiologists<br>- Nurses with experience in cardiac	Question: No change<br><br>Answer: No change

		risk prevention<br>- Paediatricians<br>- Obstetricians/ Gynaecologists<br>- Endocrinologists<br>- Others (please specify)	*This item was re-numbered to 13
15	Age for FH screening in young person	Question:<br><br>At what age would you test young individuals for hypercholesterolaemia in a family with premature CAD?<br><br>Answer:<br><br>- 0 - 6 years<br>- 7 - 12 years<br>- 13 - 18 years<br>- None of the above<br>- Don`t know	Question: No change<br><br>Answer: No change<br><br>*This items was re-numbered to 14
17	Referral to lipid specialist	Question:<br><br>If yes to question 16, have you referred patients with FH to this service?<br><br>Answer:<br><br>- Yes<br>- No<br>- Don`t know	Question: No change except for question number<br><br>If yes to question 15, have you referred patients with FH to this service?<br><br>Answer: No change<br><br>*This item was re-numbered to 16
18	Pharmacotherapy used in hypercholest-erolaemia	Question:<br><br>Which drugs do you use to treat hypercholesterolaemia? (Please select all that apply)<br><br>Answer:	Question: Rephrased<br><br>Which pharmacological agent(s) do you commonly used to treat hypercholesterolaemia? (Please select all that apply)

		- Exchange resins / bile acid sequestrants<br>- Ezetimibe<br>- Statins<br>- Fibrates<br>- Nicotinic acid<br>- None of the above<br>- Others (please specify)	Answer: No change<br><br>*This item was re-numbered to 17
19	Combined pharmacotherapy used in severe hypercholest-erolaemia (FH)	Question:<br><br>Which drug combinations do you use to treat severe hypercholesterolaemia? (Please select all that apply)<br><br>Answer:<br><br>- Statin + exchange resins / bile acid sequestrants<br>- Statin + nicotinic acid<br>- Statin + ezetimibe<br>- Statin + ezetimibe + nicotinic acid<br>- Statin + ezetimibe + exchange resins / bile acid sequestrants<br>- None of the above<br>- Others (please specify)	Question: Rephrased<br><br>Which pharmacological combination(s) do you use to treat severe hypercholesterolaemia? (Please select all that apply)<br><br>Answer: No change<br><br>*This item was re-numbered to 18
21	CAD risk stratification in FH	*Nil	New Question:<br><br>How would you risk stratify patients diagnosed with FH clinically?<br><br>Answer:

	- Perform coronary risk scoring e.g. Framingham Risk Score<br>- Classify patients as high risk irrespective of other risk factors<br>- Calculate calcium score using cardiac CT scan<br>- Determine serum hsCRP concentration<br>- Don`t know
**Total items: 9**	**Total items: 9**
